# LETTER TO THE EDITOR A New Application to Free-Nipple-Graft Reduction Mammaplasty for Breast and Nipple Projection

**Published:** 2012-08-21

**Authors:** Gamze Bektas, Anı Cinpolat, Polat Bicici, Kerim Unal, O. Koray Coskunfirat

**Affiliations:** ^a^Department of Plastic, Reconstructive and Aesthetic Surgery, Tatvan State Hospital, Bitlis, Turkey; ^b^Department of Plastic, Reconstructive and Aesthetic Surgery, Adana Numune Training and Research Hospital, Adana, Turkey; ^c^Department of Plastic, Reconstructive and Aesthetic Surgery, Akdeniz University School of Medicine, Antalya, Turkey

Dear Sir,

The free-nipple graft breast reduction is a simple and effective technique; however, inadequate breast and nipple projection are disadvantages of the technique.^1^^,^^2^ In this presentation, we aimed to apply a different modification to the free-nipple-graft reduction mammaplasty by adding nipple reconstruction with star flap and to create a good breast-nipple shape with long-lasting projection.

A 52-year-old female patient was assessed because of severe mammary hypertrophy. The distance from the sternal notch to the nipple was measured 40 cm in the right and 37 cm in the left. Free- nipple-graft reduction mammaplasty was planned. Standard-wise pattern markings were made bilaterally for skin excision. The anticipated nipple positions are marked at the level of inframammary folds. Nipples were amputated and kept as a graft bilaterally. The breast tissue pedicle was medially based and new nipple was marked on the pedicle (Fig 1). Resection is performed in the standard fashion from the inferolateral portion of the breast. A 1550-g breast tissue was excised from the right breast and 1300 g from the left breast. The pedicle was rotated toward the new location of the nipple-areola complex. Lateral and medial breast tissue sutured together to increase the breast projection. The star flaps designed with 1-cm base diameter and 1-cm height were raised on the deepithelialized pedicle (new areolar place), wrapped around the central stalk, sutured and free nipple grafts are placed directly on the flaps (Fig 1). Nipple projection was measured 1 cm intraoperatively. A tie-over dressing is applied to the grafts for 5 days.

The patient had some early wound breakdown at right areolar region, which resolved with local wound care. Follow-up period was 12 months. Nipple projection was measured 0.3 cm at the end of 12 months (Fig 2). The level of satisfaction felt by the patient was high.

Free-nipple-graft reduction mammaplasty described by Thorek^1^ is one of the procedures used to treat patients with massive breast hypertrophy. Free nipple-graft breast amputation often yields a wide, flat breast and nipple with poor projection.^2^ Several surgeons have described modifications of free nipple-graft reduction mammaplasty to solve this problem, with dermoglandular local pedicled flaps added to augment the central breast mound.^2^^,^^3^ However, these techniques could not achieve a nipple with enough projection.

We presented a free-nipple-graft technique for patients with severe hypertrophy. We formed a medial pedicle without a nipple to create an acceptable breast projection and an attractive, smooth breast contour.^4^ And, to create nipple projection, we designed a star flep on the dermoglandular medial pedicle and full-thickness nipple-areola graft is covered on top of new designed nipple.

Nipple projection was 0.3 cm at the end of 12 months; however, regression was greater than what we expected. Although projection loss had been reported 40% in nipple reconstruction with star flap in previous studies,^5^ the loss of nipple projection was 70% in our case. This difference was thought to be due to graft contraction over the deepithelialized star flap. To design flaps 3 times greater than the desired nipple was considered more appropriate for better results.

With this modified free-nipple-graft reduction mammaplasty technique, we believe that nipple projection can be sustained and much more superior aesthetic results can be obtained.

## Figures and Tables

**Figure 1:**
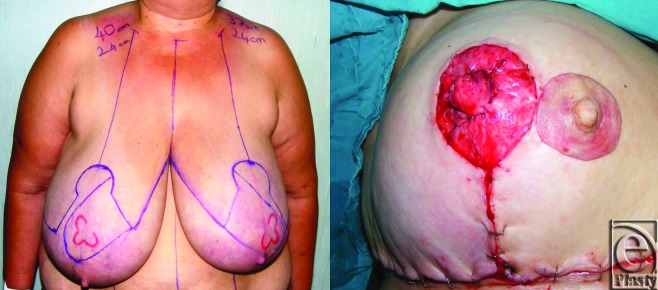
The preoperative view and intraoperative view of star flap.

**Figure 2:**
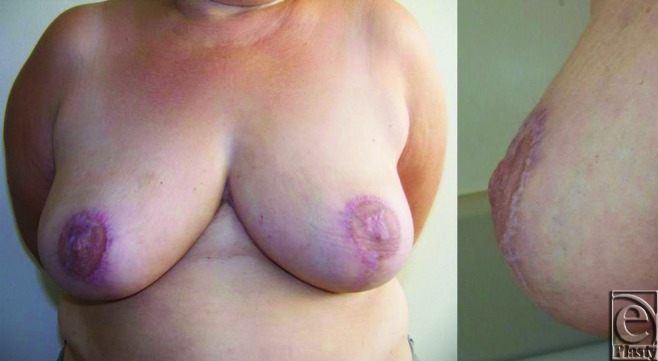
The postoperative result 1 year after the operation.
